# Association of fluid management during robotic-assisted radical laparoscopic prostatectomy with early surgical clinical outcomes: a risk factor for lymphoceles

**DOI:** 10.1007/s11701-025-02579-9

**Published:** 2025-07-22

**Authors:** Thomas Büttner, Marcus O. Thudium, Manuel Ritter, Stefan Hauser, Martin Söhle, Philipp Krausewitz

**Affiliations:** 1 Department of Urology and Pediatric Urology, University Hospital Bonn, Venusberg Campus 1, 53127 Bonn, Germany; 2 Department of Anesthesiology and Intensive Care Medicine, University Hospital Bonn, Bonn, Germany

**Keywords:** Prostatectomy, Robotic-assisted, Anesthesia, Fluid, Complications, Lymphocele

## Abstract

Robotic-assisted radical laparoscopic prostatectomy (RARP) is a standard treatment for localized prostate cancer. While surgical factors are often considered, the impact of anesthesiological factors, particularly intraoperative fluid management, on postoperative outcomes remains understudied. This study aimed to evaluate the relationship between fluid management and early complications after RARP. The study retrospectively analyzed data from 285 patients who underwent RARP at a single institution between 2019 and 2021. Fluid administration was quantified as corrected fluid dosage (mL/kg/h) and total fluid balance. Postoperative complications within 30 days, including anastomotic leakage and lymphocele formation, were assessed. Multivariable modeling and propensity score matching were used to evaluate the association between fluid management and lymphoceles. We found no significant association between fluid management and major complications (Clavien–Dindo grade ≥ II) or anastomotic leakage. However, a significant association was observed between higher fluid administration and lymphocele formation (*p* < 0.001). In the generalized linear model, the association of fluid dosage with lymphocele occurrence was confirmed (*p* = 0.002), independently of a peritoneal flap procedure. At a fluid dosage threshold of 7.73 ml/kg/h, propensity score matching confirmed the association. While major complications following RAPR are rare, generous fluid management was associated with a higher incidence of lymphocele formation. While these mostly remained asymptomatic, this finding suggests that intraoperative fluid management is a modifiable risk factor for lymphoceles and may prevent symptomatic lymphoceles as major complications in larger collectives. Moreover, it provides new insights into their potential pathogenesis.

**Supplementary Information:**

The online version contains supplementary material available at 10.1007/s11701-025-02579-9.

## Introduction

Robotic-assisted radical laparoscopic prostatectomy (RARP) represents a standard curative approach in the treatment of localized prostate cancer (PCa), the most common malignant disease in the male population of western countries [1]. Continuous improvement of RARP outcomes thus remains an ongoing challenge. While these were frequently linked with surgeon-derived factors, intraoperative factors jointly influenced by anesthesiologic and surgical decisions have been investigated far less [2–5].

The steep head-down tilt (Trendelenburg) position as well as the insufflated pneumoperitoneum require an advanced hemodynamic management [6, 7]. However, the impact of intraoperative fluid management on postoperative outcomes remains uncertain: Administering crystalloid infusions during surgery effectively increases intravascular pressure and compensates for blood loss [8]. By maintaining adequate circulation and cardiac output, intraoperative fluid management can reduce the need for catecholamines to support organ perfusion. However, the relationship between fluid administration and catecholamine requirements is complex [9]. While fluids can increase cardiac output, their effect on arterial pressure varies depending on vascular tone. In patients with low vascular tone, even significant increases in cardiac output may have minimal impact on arterial pressure, potentially necessitating vasopressor support to maintain adequate perfusion pressure. Moreover, iatrogenic fluid overload increases diffusion and loads interstitial space [10]. Hence, excessive intraoperative fluid administration can lead to tissue edema, resulting in a swollen and indistinct surgical field. This may complicate the procedure and increase the risk of postoperative complications [11, 12]. Previous meta-analyses support a fluid-sparing or goal-directed approach to reduce postoperative complications, shorten hospital stay, and facilitate bowel function recovery in oncologic surgeries [13–15]. This is consistent with Enhanced Recovery After Surgery (ERAS®) guidelines, which caution against fluid excess in elective colorectal surgery [16]. However, only a few authors have specifically explored the impact of fluid management in the context of RARP. These focused on postoperative renal and pulmonary functions, which seemed unaffected by restricted or generous fluid administration [17, 18]. Given the limited data specific to RARP outcomes beyond renal and pulmonary function, no consensus guidelines or substantiated recommendations for intraoperative fluid management in this context currently exist.

In this study, we aimed to evaluate the association of intraoperative fluid management with early postoperative complications following RARP, with a focus on outcomes of special interest, namely, anastomotic leakage and lymphocele formation. 

## Materials and methods

### Patient cohort

We screened existing datasets of patients, which underwent RARP for treatment-naïve localized PCa at a tertiary referral center (University Hospital Bonn) between 2019 and 2021. The inclusion criteria into this study comprised the full availability of baseline data, complete information on anesthesiology procedures and drug administration, as well as the availability of a complete 30-day postsurgical follow-up on outcomes. The patient selection process, including the timeframe of data collection and specific inclusion/exclusion criteria, is detailed in Supplementary Fig. 1.

### Baseline data

Demographics (age) were collected alongside clinical variables (weight, height, body mass index [BMI], American Society of Anesthesiologists [ASA] physical status classification system) and PCa data (initial PSA [iPSA], D’Amico risk group).

### Surgery

Robotic-assisted radical prostatectomy (RARP) was performed using the DaVinci® XI system following a standardized institutional protocol. All procedures were performed by one of three trained senior consultants, each with an experience of over 100 RARP procedures. Patients received antibiotic prophylaxis with 1.5 g cefuroxime administered intravenously prior to surgery, or an alternative antibiotic based on antibiogram results if preoperative urine cultures indicated bacterial growth. Anesthesia was induced using a balanced technique, combining multiple agents to achieve sedation, analgesia, and muscle relaxation. Patients were positioned in a 20° Trendelenburg position during surgery. The extent of pelvic lymph-node dissection was chosen individually, while mostly based on the D'Amico risk group (limited dissection of obturator nodes only for intermediate-risk and cN0 patients and extended pattern including internal and external iliacal nodes for high-risk or cN + patients). The margins of dissection were managed via clip-on ligatures or bipolar coagulation at the surgeons’ discretion considering the equivocal data [19]. Unilateral or bilateral nerve sparing was performed based on an individual presurgical shared-decision making. A peritoneal flap was placed at the surgeon's discretion. As per our standardized institutional protocol, no surgical drains were inserted in any patient within this cohort.

### Fluid management

We meticulously recorded all medications administered during anesthesia, including crystalloid fluids used both as standalone infusions and as solvents for other medications. To standardize fluid administration across patients, we calculated the corrected fluid dosage by dividing the total volume of fluids administered by the patient's body weight and the duration of surgery, resulting in a measurement expressed in milliliters per kilogram per hour (mL/kg/h). This weight- and time-adjusted metric is a standard measurement reported in anesthesiology literature, allowing for better comparability and reproducibility [18, 20, 21]. Additionally, we determined the total fluid balance by summing all fluid inputs—including medications dissolved in fluids—and subtracting estimated blood loss and urine output. We also recorded the mean and maximum dosages of catecholamines administered intraoperatively to assess their relationship with fluid management strategies.

### Outcomes

We collected data on any complications occurring within 30 days after RARP. Complications were graded according to the Clavien–Dindo classification, previously described in detail [22]. Briefly, grade I covers any deviation from normal postsurgical course without a need for treatment beyond supportive drugs. Grade II includes any pharmacological treatment beyond these. Grade III includes interventional or surgical re-treatment without (IIIa) or with (IIIb) general anesthesia. Grade IV describes organ dysfunction, of a single organ (IVa) or multi-organ failure (IVb). Grade V marks a lethal complication.

We defined two complications of special interest in the early post-RAPR setting: leakage of the vesicourethral anastomosis and the formation of a pelvic lymphocele, routinely assessed in all patients: At removal of the indwelling transurethral catheter, cystography was performed at the surgeon’s discretion to confirm the integrity of the anastomosis. The formation of lymphoceles was checked in all patients by ultrasonography of pelvic vessels on day 6 after surgery regardless of the presence or absence of associated symptoms.

### Confounder adjustment

For any outcomes significantly associated with fluid dosage, adjustments were performed to account for confounders. The following variables were fitted into a multivariable generalized linear model for the binominal dependent outcome: age, ASA score (1–2 vs. ≥ 3), extend of lymph-node resection (extended vs. limited), peritoneal flap, duration of surgery, mean noradrenaline dosage, and corrected crystalloid fluid dosage.

To gain further insight and confirm potential relationships, the optimal threshold of corrected crystalloid fluid dosage to predict the outcome was calculated to define groups with either low or high fluid input. Next, propensity scores were generated for the variables mentioned above. Thereafter, we performed a propensity weighted matching of patients who from either low or high fluid dosage groups and compared the outcome rates between these groups.

### Statistics

Descriptive statistics included frequencies and proportions for categorical variables. Medians and interquartile ranges (IQR) were reported for continuously coded variables. Differences and relations were assessed by Wilcoxon rang sum tests and Fisher’s exact tests with odds ratio (OR) and 95% confidence interval (95% CI). Risk factors for both complications of special interest (anastomotic leakage and pelvic lymphocele) were fitted into multivariable generalized linear models, selected with respect to potential collinearity. The Youden-Index in Receiver-Operating Characteristics (ROC) was used to determine the best cut-off value. Propensity scores were generated based on logistic regression and matching was performed by the “*optimal*” method [23].

All data were coded and analyzed using RStudio 2024.09.0 + 375 (https://CRAN.R-project.org/) using the R packages *pROC* (v1.18.5) *MatchIt* (v4.6.0), *ggplot2* (v3.5.1), and *ggpubr* (v0.6.0).

## Results

**Fig. 1:**
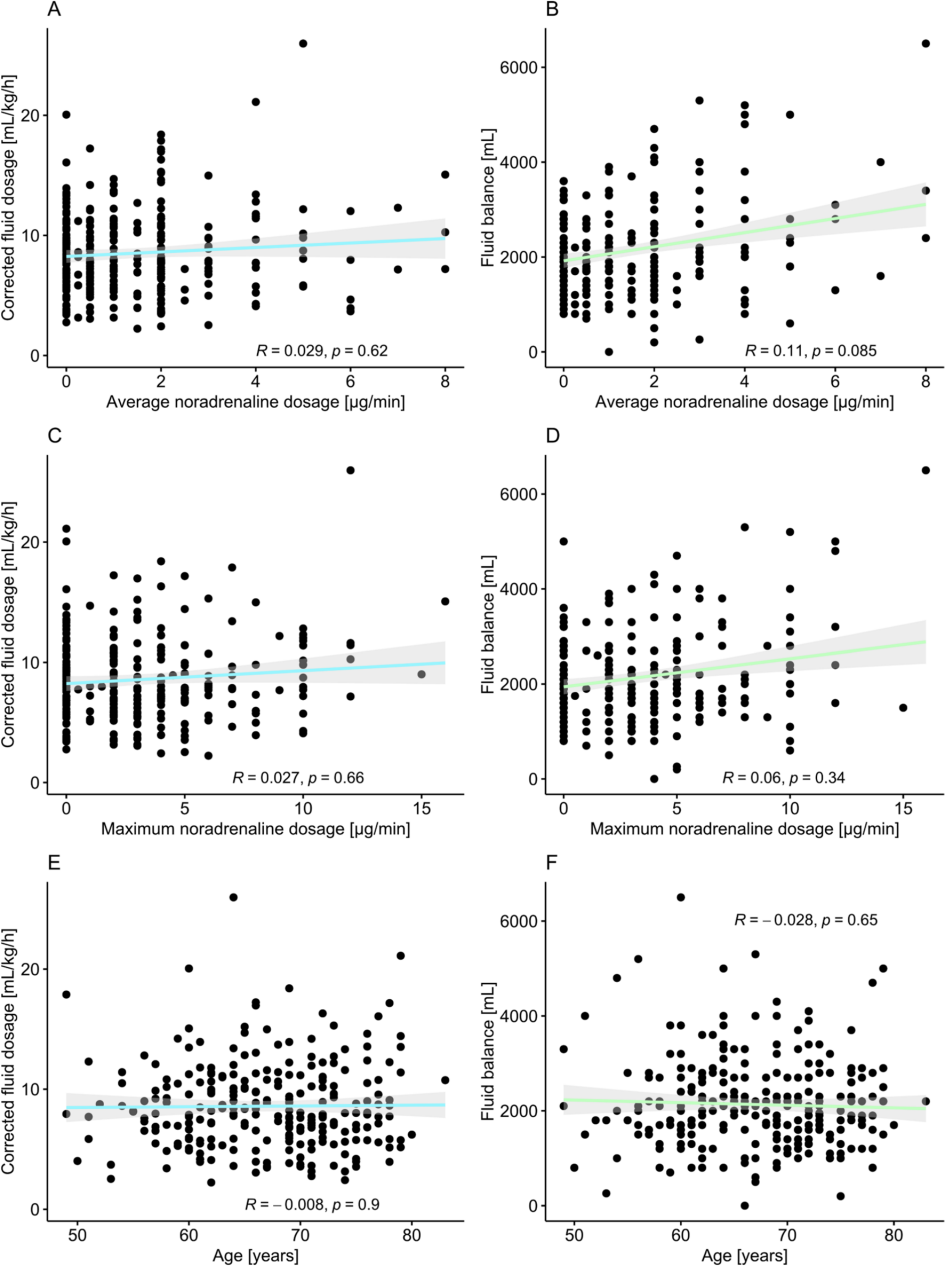
Scatterplots and correlation analysis of average (**A + C + E**) and maximum (**B + D + F**) noradrenaline dosage with corrected crystalloid fluid dosage (**A + B**), fluid balance (**C + D**), and age (**E + F**). No significance or meaningful correlation was found (all *p* > 0.05 and Spearman’s *R* < 0.1)

*N* = 285 men met the selection criteria and were included into the analysis. The cohort represented a typical cohort of localized PCa patients scheduled for curative surgery, marked by a median age of 67 years, median BMI of 26.4 kg/m^2^, and mostly favorable ASA score (1–2 in 81.8% of patients) and D’Amico risk group (low and intermediate risk in 69.8%). Median duration of surgery was 189 min. During this period, we administered a median corrected fluid dosage of 8.0 mL/kg/h. The overall fluid balance, compromising any inputs and losses, was at a median of 2000 mL. 237 (86.2%) of patients required catecholamines, with a median average noradrenaline dosage of 1.0 µg/min and a median maximum dosage of 2.0 µg/min. Neither average nor maximum noradrenaline dosage was associated with fluid administration or balance (average dosage: Fig. 1 A + B, Spearman’s *R* = 0.03, *p* = 0.62 and *R* = 0.08, *p* = 0.17, respectively, and maximum dosage: Fig. 1 B + C, *R* = 0.02, *p* = 0.71 and *R* = 0.05, *p* = 0.45, respectively). We dissected a limited lymph-node pattern in the majority of patients and performed a peritoneal flap in about half of the cohort (68.9% and 50.9%, respectively). All baseline and surgery characteristics are depicted in Table 1.

**Table 1:** Baseline characteristics

Parameter	Overall, *N* = 285 Median [IQR] or *N* (%)
Age (years)	67 [62, 72]
BMI (kg/m^2^)	26.4 [24.3, 28.7]
iPSA (ng/mL)	7.6 [5.5, 10.9]
ASA score	
1	40 (14.0%)
2	194 (68.1%)
3	51 (17.9%)
D’Amico risk group	
Low risk	33 (11.6%)
Intermediate risk	165 (57.9%)
High risk	86 (30.2%)
*Missing*	1 (0.4%)
Lymph-node dissection	
Limited	199 (69.8%)
Extended	86 (30.2%)
Lymph nodes dissected (count*)*	9 [6, 13]
Prostate weight (g)	46.1 [38.0, 62.8]
Nerve sparing	
Yes, bilateral	86 (30.2%)
Yes, unilateral	70 (24.6%)
No	130 (45.6%)
Peritoneal flap	
Yes	140 (49.1%)
No	136 (47.7%)
*Missing*	9 (3.2%)
Blood loss (mL)	400 [250, 500]
Duration of surgery (min)	189 [168, 213]
Average noradrenaline dosage (µg/min)	1.0 [0.5, 2.0]
Maximum noradrenaline dosage (µg/min)	2.0 [0.5, 5.0]
Total crystalloid fluids during surgery (mL)	2,000 [1,500, 2,500]
Corrected fluid dosage (mL/kg/h)	8.0 [6.0, 10.6]
Fluid balance (all inputs – all losses, mL)	+ 2,000 [+ 1,600, + 2,700]

*BMI* Body mass index, *PSA* Prostate-specific antigen, *ASA* American Society of Anesthesiologists physical status classification system

### Overall complications

**Fig. 2:**
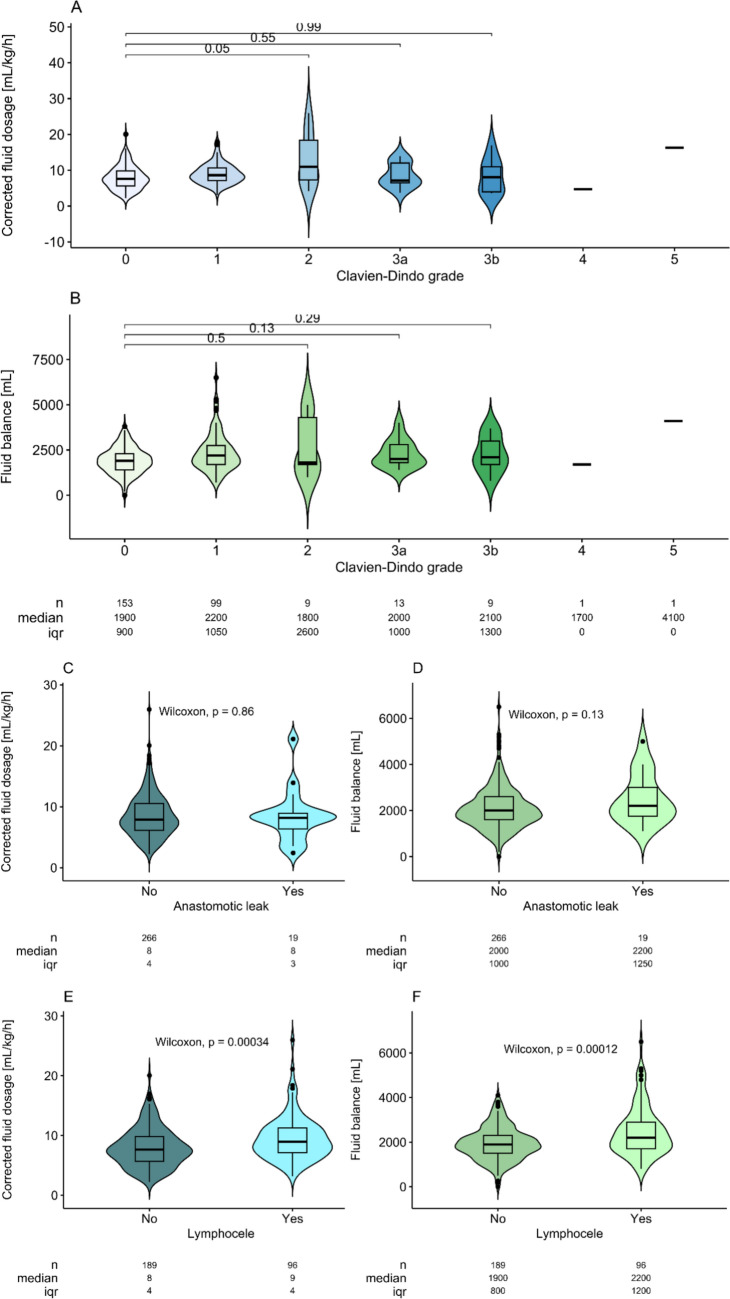
Violin–Boxplots of corrected crystalloid fluid dosage (**A, C, E**) and fluid balance (**B, D, F**) during surgery as a risk factor for postsurgical events. Clavien–Dindo grade > 1 complication (**A + B**) and anastomotic leakage (**C + D**) were not significantly associated with fluid management (*p* > 0.05 all). In contrast, lymphocele occurrence was strongly associated with both fluid management parameters (**E + F**, *p* < 0.001 both)

During the 30 days following surgery, 132 patients (46.3%) experienced complications. 99 (34.7%) were Clavien–Dindo grade I complications, while 33 (11.6%) suffered from clinically relevant complications ≥ Clavien–Dindo grade II including one fatal grade V complication of pulmonary embolism, one grade IVa complication of delirium, and 9 (3.3%) grade IIIb complications requiring a re-intervention under anesthesia. Table 2 sums up the rates. Neither corrected fluid administration nor fluid balance was associated with the clinically relevant complications of grade II, IIIa, or IIIb (Fig. 2 A + B, corrected: *p* = 0.05, *p* = 0.55, and *p* = 0.99, balance: *p* = 0.50, *p* = 0.13, and *p* = 0.29, respectively). Higher graded complications were not analyzed due to their rarity.

**Table 2:** Complication outcomes

Parameter	Overall *N* = 285
Complications by Clavien–Dindo grade *N* (%)	
0	153 (53.7%)
I	99 (34.7%)
II	9 (3.2%)
IIIa	13 (4.6%)
IIIb	9 (3.2%)
IVa	1 (0.4%)
IVb	0 (0.0%)
V	1 (0.4%)
Complications of special interest *N* (%)	
Anastomotic leakage	16 (6.7%)
Lymphocele	93 (33.7%)
Asymptomatic	86 (30.2%)
Symptomatic	7 (2.5%)

### Anastomotic leakage

Nineteen patients (6.7%) developed a cryptographically confirmed anastomotic leakage, leading to a delayed removal of transurethral catheter (Table 2). Fluid management was not associated with this outcome (Fig. 2 C + D, *p* = 0.86 for corrected fluid dosage and *p* = 0.13 for fluid balance).

### Lymphoceles

About one-third of the cohort (*N* = 93, 33.7%) of patients developed a pelvic lymphocele, commonly as an asymptomatic routine finding in 92.5% of these (*N* = 86; Table 2). Crystalloid fluid dosage as well as fluid balance were significantly associated with a postsurgical lymphocele occurrence (Fig. 2 E + F, *p* < 0.001 both). However, only 7 of the 93 lymphoceles (7.5%) were clinically symptomatic and requiring an intervention (drainage ± antibiotics). This group size was too small for statistical significance testing; median corrected fluid dosage was 8.4 mL/kg/h [IQR: 7.1–10.8] in these seven patients compared to 7.9 mL/kg/h [IQR: 6.0–10.5] in all remaining. 

### Cofounder adjustment

In the generalized linear model, the association of fluid dosage with lymphoceles was confirmed after adjusting for covariables (estimate: 0.132, *p* = 0.002). Also, the formation of a peritoneal flap was significantly associated with a reduced rate in this model (estimate: − 0.574, *p* = 0.042). None of the further included parameters displayed a significant association with lymphocele occurrence in this analysis (Table 3).

**Table 3:** Multivariable generalized linear model of potential risk factors for lymphocele development

Overall cohort (*N* = 285)		
Parameter	Estimate	*p *value
Age (years)	− 0.026	0.177
ASA Score (1–2 vs. 3)	0.305	0.413
Lymph-node dissection (extended vs. limited)	0.411	0.125
Peritoneal flap (yes vs. no)	− 0.574	0.042*
Duration of surgery (min)	0.002	0.589
Average noradrenaline dosage (µg/h)	0.004	0.956
Corrected fluid dosage (mL/kg/h)	0.132	0.002*

^*ASA*^ American Society of Anesthesiologists physical status classification system

^*^^p^^ < 0.05^

^**^^p^^ < 0.01^

In ROC analysis, the optimal corrected crystalloid fluid dosage threshold to predict lymphocele formation was at 7.73 ml/kg/h. Looking at the 154 patients with a high fluid dosage according to this cut-off, lymphoceles occurred in 66 (42.9%), contrasting 30 (22.9%) in the remaining 131 men (OR: 2.52, 95% CI: 1.46–4.40; *p* < 0.001). After propensity matching, each group comprised 122 patients. Distribution of propensity scores is displayed in Supplementary Fig. 2. In the high fluid dosage group, 55 (45.1%) developed lymphoceles compared to 28 (23.0%) in the low fluid dosage group (OR: 2.74, 95% CI: 1.53–4.99; *p* < 0.001, Table 4).

**Table 4:** Lymphocele rates in the overall cohort and the propensity matched cohort by corrected fluid dosage at a cut-off of 7.73 ml/kg/h (high vs. low dosage)

Overall cohort (*N* = 285)				
Fluid dosage	Low (*N* = 131)	High (*N* = 154)	OR (95% CI)^1^	*p* value^1^
Lymphocele (*N*, %)	30 (22.9%)	66 (42.9%)	2.52 (1.46–4.40)	< 0.001***

Matched cohort (*N* = 244)				
Fluid dosage	Low (*N* = 122)	High (*N* = 122)	OR (95% CI)	*p* value
Lymphocele (*N*, %)	28 (23.0%)	55 (45.1%)	2.74 (1.53–4.99)	< 0.001***

^*OR*^ odds ratio, *95% CI* 95% confidence interval

^1Fisher^^’s exact test^

^***^^p^^ < 0.001^

## Discussion

For the first time, our study unveiled an association of lymphocele formation with fluid management during RARP. An equally important finding is the absence of an association with the occurrence of anastomotic leakage or complications Clavien–Dindo grade ≥ II. Hence, our findings offer a comprehensive insight with implications for clinical practice.

There is still an ingrained perception among many surgeons that excessive fluid management will contribute to complications. This may simply be a subjective bias, induced through an increased fluid usage in prolonged and challenging cases, or that it is difficult to imagine how edematous tissue and anastomosis will successfully recover [24]. In a meta-analysis of randomized-controlled trials, the rate of surgical complications was decreased by about 10% in patients receiving goal-directed fluids compared to liberal perioperative fluid strategies. Still, mortality and rates of complications involving major organ systems, such as cardiac, pulmonary, renal, or central nervous disorders, were not significantly affected [25]. Our results can be interpreted as consistent with these findings. It must be noted though that severe complications are rare within the perioperative course of RARP [26]. Hence, larger scaled studies are needed to draw final conclusions on complications Clavien–Dindo grade ≥ II. However, if mild complications are taken into account, a possible role of fluid management in prevention of complications emerges.

Our results suggest that lymphoceles more frequently occur following a generous fluid management, either in corrected crystalloid fluid dosage or overall fluid balance. Still, concerns can be kept within reasonable limits: the vast majority of these were asymptomatic and did not require intervention, leading to a classification of Clavien–Dindo grade I complications. As with any retrospective analysis, the possibility of residual confounding influencing this association cannot be fully excluded. The significant association, even after adjustment for covariates in the generalized linear model, still supports the hypothesis that fluid management may represent an independent, associative risk factor. Therefore, our findings provide a potential explanation and a novel step toward an understanding on the formation of lymphoceles, a complication which remains to be fully understood to date [27]. Furthermore, understanding the relationship between intraoperative fluid management and lymphocele formation could inform strategies to minimize this risk.

Lymphoceles are pathogenically supported by surgically induced leakage of lymphatic fluid, exacerbated by a lack of supporting tissue after dissection [28]. Crystalloid fluid administration during surgery increases lymphatic flow by a large extend [29]. Excessive fluid management may therefore result in increased lymphatic fluid production and thus maintain microleakage of the disrupted lymphatic vessels. Additionally, crystalloid fluid administration significantly lowers colloid osmotic pressure (COP), and lower COP has been linked to lymphocele development [30, 31]. Either of these mechanisms or an additive effect may provide a hypothetic explanation for our results.

No prior studies have reported any findings regarding the association, or lack thereof, between this topic and related outcomes. A correlation with the extent of lymphadenectomy, BMI, and age has been repeatedly described before, therefore representing potential risk factors, although especially data on age and BMI remain inconsistent [27, 32–36]. In our analysis, we found a non-significant trend toward lymphocele formation following an extended lymph-node dissection (estimate: 0.411, *p* = 0.125), potentially supporting these findings while possibly relying on a cohort underpowered to detect significant differences in this respect. Age displayed only negligible associations if adjusted for covariables, while BMI could not be included into the model alongside the weight-corrected fluid dosage due to collinearity. However, these mentioned variables are often unmodifiable determined by the oncological stage and patient condition. We identify a modifiable potential risk factor and propose the first potential protective measure against lymphocele development, which is primarily influenced by the anesthesiologist. Considering the encouraging evidence for the potential of individualized fluid management, our result supports further evaluation in the context of RARP to avoid postoperative complications and improve outcomes [13, 14].

Since fluid dosage remained significantly related to lymphocele rates in both the generalized linear model and the confirmatory propensity matched cohort, we strongly believe that the proposed association may present an independent risk factor. However, potential confounders cannot be omitted, as the underlying motives cannot be specified due to the retrospective nature of the data. Catecholamine dosage was not correlated to fluid dosage; hence, it can be concluded that higher dosages were not exclusively administered to patients with an unstable circulation during surgery. And considering crystalloid fluid administration is rather ineffective to maintain blood pressure and cardiac output [37], our findings suggest that clinicians might consider a more restrictive fluid strategy, potentially aiming for a dosage below the identified threshold of 7.73 ml/kg/h. In the absence of an association of noradrenaline dosage and lymphocele occurrence, this strategy to improve circulation may be preferable in this regard. Given the low clinical impact of most observed lymphoceles, this should remain a clinical consideration balanced against the patient's overall hemodynamic status, rather than a strict recommendation.

This approach becomes particularly relevant when looking at the limited options that can be influenced by the surgeon. Only a peritoneal flap has been convincingly investigated in this context. Most prominently, the large-scale RCTs *ProLy* and *PELYCAN* demonstrated lower rates of lymphoceles in the peritoneal flap arm [38–40]. Our observations confirmed this, with a peritoneal flap reducing the estimated probability of a lymphocele by over one half after adjustment for covariates (GLM estimate: − 0.574). Our cohort of 285 men was considerably smaller in contrast to these trials (*ProLy*:* N* = 530, *PELYCAN*:* N* = 551), and our retrospective design will likely lead to more imbalance between patients with or without peritoneal flap compared to an RCT. Nevertheless, our approximately one-third rate of lymphoceles as well as the risk reduction with a peritoneal flap was comparable to that reported in these trials (*ProLy*: 33% without peritoneal flap and 22% with peritoneal flap, *PELYCAN*: 27.2% without peritoneal flap and 10.3% with peritoneal flap). Unfortunately, details of intraoperative fluid management were not reported in these RCTs, so site-specific variations in this regard can be hypothesized, but ultimately not explored. Importantly, as in the *PELYCAN* and *ProLy* trials, the vast majority of lymphoceles in our cohort remained asymptomatic [38, 40]. However, since there is a correlation between the overall occurrence and the rate of symptomatic lymphoceles, we assume that avoiding risk factors for overall lymphoceles will also reduce the amount of symptomatic cases [39].

### Limitations

Patients followed an institutional standardized procedure. Therefore, findings may potentially not be transferrable into settings significantly diverging from our surgical management. External validation of the outlined associations is warranted. 30-day follow-up is insufficient to report on findings regarding long-term outcomes, especially continence and sexual function. Major complications were rare; therefore, the cohort size may not have been powered to detect any significant differences based on fluid management here. Furthermore, the reliance on ultrasonography on a fixed postoperative day (day 6) for lymphocele detection likely underestimates the true incidence, as some may develop or become clinically apparent at a later time [19]. Our analysis was focused on intraoperative factors and did not include data on postoperative fluid management. Although routine intravenous fluids are not part of our standard postoperative care, the absence of these data is a limitation, as fluid balance in the immediate postoperative days could also influence lymphocele development.

## Conclusions

Fluid management during RARP was not found to be associated with major complications in our cohort. However, lymphocele formation was linked to more generous fluid management, either through corrected fluid dosage or overall fluid balance. These findings offer new insights into the pathogenesis of lymphoceles and highlight a potentially modifiable risk factor related to anesthesia in PCa surgery.

## Supplementary Information

Below is the link to the electronic supplementary material.

Supplementary file1 (DOCX 107 KB)

## Data Availability

The data that support the findings of this study are available from the corresponding author (Thomas.Buettner@ukbonn.de) upon reasonable request.
