# Supplementary material for: Association of fluid management during robotic-assisted radical laparoscopic prostatectomy with early surgical clinical outcomes: a risk factor for lymphoceles
Source: J Robot Surg. 2025 Jul 22;19(1):413. doi: 10.1007/s11701-025-02579-9 (PMC12283430; doi:10.1007/s11701-025-02579-9)
Supplement: Supplementary file 1 — Supplementary file1 (DOCX 107 KB) [file 11701_2025_2579_MOESM1_ESM.docx]

**Supplementary files**

**Supplementary Figure 1:** Study flow diagram


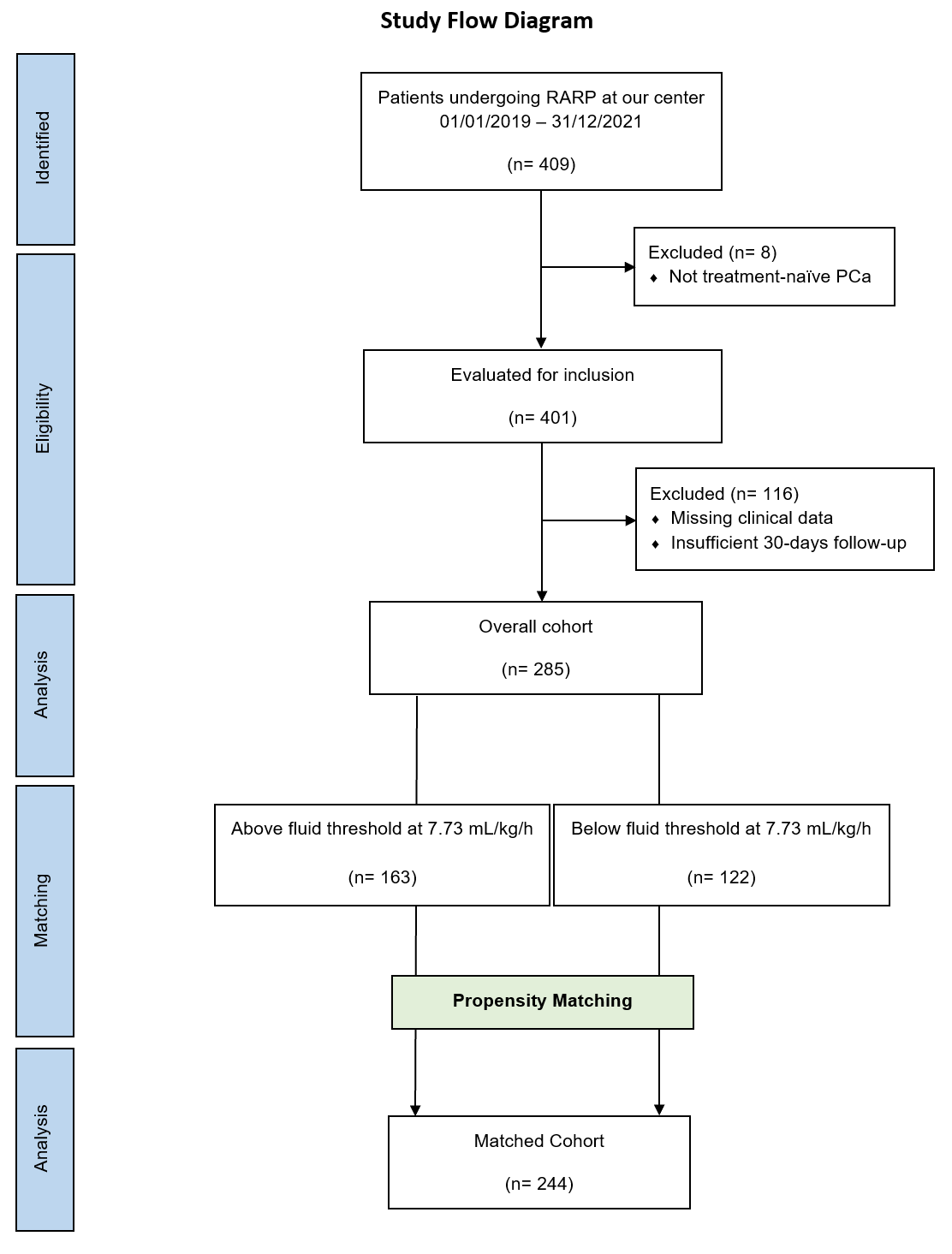


**Supplementary Figure 2:** Distribution of Propensity scores

***~~
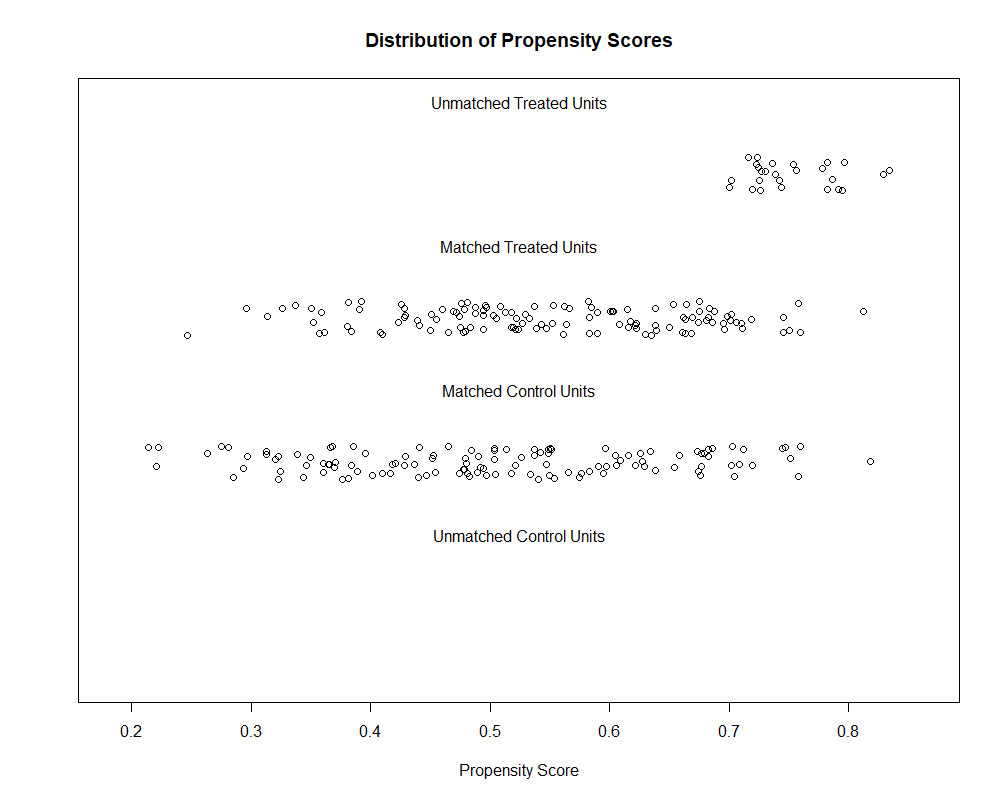
~~***
